# Editor’s introduction to this issue (G&I 19:1, 2021)

**DOI:** 10.5808/gi.21191

**Published:** 2021-03-29

**Authors:** Taesung Park

**Affiliations:** Department of Statistics, Seoul National University, Seoul 08826, Korea

In this issue, there are 10 Original Articles, of which five are related to cancer research. The first original article, by the group of Han et al. (Ewha Womans University, Korea), focused on elucidating the molecular mechanisms of acquired resistance to BRAF inhibitors in melanoma. A microfluidic chip with a concentration gradient of vemurafenib was utilized to rapidly obtain therapy-resistant clones from two melanoma cell lines with the *BRAF^V600E^* mutation. Exome and transcriptome data were produced from 13 resistant clones. This study provides an omics-based comprehensive overview of the molecular mechanisms governing acquired resistance to BRAF inhibitor therapy. The second article, by Lee and Jung (KAIST, Korea), reported functional annotation of lung cancer-associated genetic variants based on eight major cell types of human lung tissue. This work showed that approximately 22% of lung cancer-associated risk variants were linked to noncoding regulatory elements. Through integrative analysis of high-resolution long-range chromatin interactome maps and single-cell RNA-sequencing data, the authors uncovered a number of putative target genes of these variants and functionally relevant cell types, which expands the scope of functional annotation of lung cancer-associated genetic risk factors.

The third article, by Mathavan et al. (Management and Science University, Malaysia), identified potential candidate genes for lip and oral cavity cancer using network analysis. Using the DisGeNET database and STRING database, the authors identified several hub genes, such as *VEGFA*, *IL6*, *MAPK3*, *INS*, *TNF*, *MAPK8*, *MMP9*, *CXCL8*, *EGF*, and *PTGS2*, which could provide a new understanding of the underlying molecular mechanisms of lip and oral cavity cancer. The fourth article, by Jain et al. (Saveetha Institute of Medical and Technical Sciences, India), reported genetic alterations in the WNT family of genes and their putative association with head and neck squamous cell carcinoma. The WNT signaling pathway is known to be involved in crucial mechanisms for cellular maintenance and development. The authors reported a marked difference in the gene expression profile of *WNT11* between grades and when compared with normal samples. The fifth article, by Shahik et al. (AFC Agro Biotech Ltd., Bangladesh), presented the results of screening alkaloid inhibitors for vascular endothelial growth factor (VEGF) in cancer cells. Through an integrated computational approach, the authors proposed five alkaloid candidates for inhibiting VEGF and VEGR receptor‒mediated angiogenesis, which can be used as novel lead compounds to design new and effective drugs against cancer.

The sixth article, by Rath et al. (Odisha University of Agriculture and Technology, India), investigated the in silico discovery and evaluation of phytochemicals of the plant *Withania somnifera*. As a putative bioenhancer of levodopa therapy in Parkinson disease, the authors reported nine phytochemicals that had strong binding efficiency against human catechol-O-methyltransferase in comparison to the inhibitory drugs opicapone and entacapone. The seventh article, by Ponnanna et al. (University of Mysore, India) reported de novo assembly, annotation, and gene expression profiles of gonads of Cytorace-3, a hybrid lineage of *Drosophila nasuta nasuta* and *D. n. albomicans*. This study provided an overview of the expression divergence and inheritance patterns of transcriptomes in an independently evolving distinct hybrid lineage of *Drosophila*. The next article, by Oh (Inje University College of Medicine, Korea), performed a computational evaluation of interactions between olfactory receptor olfactory receptor 2W1 (OR2W1) and its ligands. Through modeling the interaction between an olfactory receptor and its ligands at the molecular level, the author successfully demonstrated the modes of ligands binding to the three-dimensional (3D) model of olfactory receptor OR2W1 and showed a statistically significant difference in the binding affinity to the olfactory receptor between the agonist and the antagonist.

The last two articles are about machine learning and statistical modeling. The article by Qiu et al. (Dankook University, Korea) established machine learning-based prediction models of anti-cancer drug response using cancer cell line gene expression and drug response data. Several statistical methods, such as Pearson correlation analysis and an ElasticNet regression model, were performed to find the model with the best performance. The last article, by Goo et al. (Seoul National University, Korea), is about predicting the future spread of coronavirus disease 2019 (COVID-19) in Korea. Using five mathematical, machine learning, and statistical models, the authors predicted the daily number of COVID-19 confirmed cases. Through comparative studies, the authors showed that machine learning models, a standard susceptible exposed infected recoverd model, and a non-linear model tended to provide accurate predictions. These prediction results are expected to help in the pandemic response by informing decisions about planning, resource allocation, and decisions concerning social distancing policies.

